# The Neural Correlates of Access Consciousness and Phenomenal Consciousness Seem to Coincide and Would Correspond to a Memory Center, an Activation Center and Eight Parallel Convergence Centers

**DOI:** 10.3389/fpsyg.2021.749610

**Published:** 2021-09-29

**Authors:** Giancarlo Frigato

**Affiliations:** Department of General Psychology, University of Padua, Padua, Italy

**Keywords:** attention, access consciousness, phenomenal consciousness, points of convergence, animal consciousness

## Abstract

An increasing number of authors suggest that the neural correlates of consciousness (NCC) have no selective, executive, or metacognitive function. It is believed that attention unconsciously selects the contents that will become conscious. Consciousness would have only the fundamental function of transforming the selected contents into a format easily used by high-level processors, such as working memory, language, or autobiographical memory. According to Dehaene, the neural correlates (NC) of *access consciousness* (AC; cognitive consciousness) constitute a widespread network in the frontal, parietal, and temporal cortices. While Tononi localized the correlates of *phenomenal consciousness* (PC; subjective consciousness) to a posterior “hot zone” in the temporo-parietal cortex. A careful examination of the works of these two groups leads to the conclusion that the correlates of access and PC coincide. The two consciousnesses are therefore two faces of the same single consciousness with both its cognitive and subjective contents. A review of the literature of the pathology called “neglect” confirms that the common correlates include 10: a memory center, an activation center, and eight parallel centers. From study of the “imagery” it can be deduced that these eight parallel centers would operate as points of convergence in the third person linking the respective eight sensory-motor-emotional areas activated by external perceptions and the corresponding memories of these perceptions deposited in the memory center. The first four centers of convergence appear in the most evolved fish and gradually reach eight in humans.

## Introduction

In the current state of the field, the consensus is increasing in favor of the hypothesis that there is a clear separation between attention and consciousness (van Boxtel et al., 2010; Cohen et al., 2012; Koch and Tsuchiya, 2015; Baier et al., 2020; Davidson et al., 2020). In this context, attention is believed to select from among the unconscious perceptual images coming from either the environment or memory, those that are then made conscious by the neural correlates of consciousness (NCC; Mashour et al., 2020). To support this, based largely on studies on decision-making, hypnosis, or “neglect,” several authors agree that consciousness has no selective, executive, control, or metacognitive function (Libet et al., 1983; Velmans, 2000; Earl, 2014, 2019; Oakley and Halligan, 2017). It instead, has the exclusive ability to provide content in a format that constitutes the only source usable by high-level processors such as working memory, the decision-making executive and metacognition centers, and the language or memories of conscious contents (Earl, 2014; Newell and Shanks, 2014; Jacobs and Silvanto, 2015; Oakley and Halligan, 2017; Kanai et al., 2019; Mashour et al., 2020). Such processors that act through unconscious mechanisms consequently produce additional unconscious contents which in turn, will be made conscious by the NCC (Jacobs and Silvanto, 2015; Persuh et al., 2018). These are then further available for new elaborations and so on, creating a continuous flow of consciousness (James, 1906).

Group of Dehaene originally postulated that consciousness was directly involved in the operational mechanism of attention, working memory (Dehaene and Changeux, 2004), or metacognition (Dehaene et al., 2017); however, they now appear convinced of only an indirect involvement of consciousness in such mechanisms, even if they maintain a certain ambiguity in this regard (Mashour et al., 2020). It is also useful to recall the well-known distinction between access consciousness (AC) and phenomenal consciousness (PC; Block, 1995). The AC (“easy problem,” Chalmers, 1996) refers to the cognitive contents of consciousness that can precisely be used by high-level processors. The PC (“hard problem,” Chalmers, 1996) refers instead to subjective sensations that cannot be clearly defined, relating for example, to colors, sounds, flavors, or emotions that we experience in our conscious life (aka, the “qualia”). In the following sections, I make an extensive summary to allow the reader to more easily follow the various passages of the theory set out in this article.

### The Neural Correlates of Consciousness

The NCC problem is considered of fundamental importance and has been the most extensively studied. Identification of the NCC and the mechanisms through which they make conscious the perceptions selected by attention, provides useful information to attempt to solve some other problems of consciousness. In identifying NCC, important experimental and theoretical results have been obtained which apparently vary from one research group to another. In particular, there are two main theories proposed for the identity of the NCC. First, the global neuronal workspace theory (GNWT) of the group of Dehaene and Changeux (2004), inspired by the theory of Baars (1988), which is dedicated to the research of the neural correlates (NC) of AC. Second, the Integrated Information Theory (IIT) by Tononi (Boly et al., 2017), inspired by Edelman (1989), which instead refers to the NC of PC.

By comparing the NC of the AC that have been identified by Dehaene’s group with those of the NC of the PC identified by Tononi’s group, and taking into account the necessary criticisms and considerations, it is possible to characterize the NCC common to the two groups. Using this approach, it becomes clear that the real difference between the two theories concerns only the involvement or not of the frontal lobe, not in its entirety, but for the dorsolateral prefrontal part, Broadmann areas (BA) 9/10/11/12. Considering that the dorsolateral prefrontal part of the frontal lobe appears active only in the post-consciousness phase, then it follows that the NC of the AC seem to coincide with the NC of the PC. As a result, AC and PC would therefore be two faces of the same coin, that is, of the one consciousness. With the cognitive contents inseparable from the subjective ones.

To substantiate this hypothesis, I will reference my “neglect” review study (Frigato, 2014). As is known, “neglect” is a pathology due to unilateral lesions generally affecting the right side of the brain (Lunven and Bartolomeo, 2017). It is characterized by the complete loss of consciousness, and therefore, the inability to report what happens in the left side of the perceptual space (Vallar, 1998, 2007) or in the imagination (Bisiach and Luzzatti, 1978). We will see that a thorough study of neglect confirms, from a neuropsychological point of view, that precisely the 10 NCC common to GNW and IIT are in fact, the NC of the unique consciousness. The 10 NCC would be: anterior dorsal cingulate (BA 32), posterior cingulate-retrosplenial-precuneus (BA 23/7/31), superior temporal lobe (BA 22/37), superior parietal lobe (BA 7a), premotor area BA 6 and premotor BA 8, anterior insula, posterior insula, inferior frontal lobe (BA 44/45/46/47), and inferior parietal lobe (BA 39/40; See Table 1).

**Table 1:** Ten neural correlates of consciousness.

Brain region (BA)	Theory	Functional role
Anterior dorsal cingulate (BA 32)	GNWT (IIT)	Activation center of the 8 parallel convergence centers
Posterior cingulate-retrosplenial-precuneus (BA 23/7/31)	GNWT-IIT	Memory center
Superior temporal lobe (BA 22/37)	GNWT-IIT	“What” is perceived
Superior parietal lobe (BA 7A)	GNWT-IIT	“Where” perception is positioned
Premotor area (BA 6)	GNWT (IIT)	Feeling of being the Actor of one’s own peripersonal motor activities related to perception
Premotor area (BA 8)	GNWT (IIT)	Feeling of being the Actor of one’s own extrapersonal motor activities related to perception
Anterior insula	GNWT (IIT)	Emotions connected to perception
Posterior insula	GNWT (IIT)	Feeling of owning one’s own body during perception
Inferior frontal lobe (BA 44/45/46/47)	GNWT (IIT)	Feeling of being the Author in the Cartesian theater of perception
Inferior parietal lobe (BA 39/40)	GNWT-IIT	Feeling of being the Spectator in the Cartesian theater of perception

GNWT=neural correlates of consciousness (NCC) identified by the Dehaene group. IIT=NCC identified by the Tononi group. (IIT)=NCC identified by the Tononi group, but not taken into consideration as their bilateral lesion does not cause rogue loss of consciousness (Boly et al., 2017).

The posterior cingulate-retrosplenial-precuneus cortex would constitute the complex of memories of conscious contents (Damasio, 1999; Vogt and Laureys, 2005; Cavanna and Trimble, 2006; Burianova and Grady, 2007; Cavanna, 2007; Rolls, 2019). While the anterior dorsal cingulate would have the task of simultaneously activating the other eight NCC for the time necessary (about 100–150ms) to produce conscious contents (Cairns et al., 1941; Laplane et al., 1981; Posner and Dehaene, 1994; Damasio, 1999; Bush, 2011; Rolls, 2019). The hippocampus would in turn, deposit such contents in the complex of memories (Squire and Schacter, 2002).

The eight NCC would constitute eight different types of functional centers in the cerebral cortex that operate in parallel, closely linked and coordinated with each other, and with the ability to produce eight different contents. These contents relate to: “what” is perceived (BA 22/37); “where” perception is positioned (BA 7a); feelings of self-agent and their peripersonal (BA 6) and extrapersonal (BA 8) motor activities related to this perception; emotions connected to it (insula ant.); perception of one’s body at that moment (insula post.); feeling of being the author (BA 44/45/46/47) and at the same time spectator (BA 39/40) in the Cartesian theater (Baars, 1997) of this perception. From time to time, one of the eight contents predominates and the others remain in the background. For example, the characteristics of an object may predominate, i.e., the “what,” while the other seven contents are contained simultaneously as accompaniment.

We will see that these eight NCC coincide with the eight NC that are equally active in both external perception and “imagery.” Taking a cue from this consideration, we arrive at the hypothesis that the eight NCC function as “centers of convergence in the third person” (Damasio, 1994, 1999) which, through reentrant connections (Edelman, 1989; Lamme, 2018), keep active and united the brain areas responsible for specific external sensory-motor-emotional perceptions together with the corresponding memories, positioned precisely in the complex of memories. These in turn, may be the sources of imagery produced by these perceptions (Ganis et al., 2004).

## Neural Correlates Equal for Access Consciousness and Phenomenal Consciousness

The research summarized and interpreted here on the NC of the AC and the PC are made as precisely and objectively as possible, but the investigation techniques and the study of neglect obviously can lead to errors and omissions. For example, some identified area could actually be part of a larger complex. However, while taking this into account, to simplify the reading and understanding of the concepts set out here, 10 very specific and delimited areas will be considered that reasonably seem to be the NC responsible for consciousness.

*The group of Dehaene and Changeux* put forward the GNWT (2004, 2011; Mashour et al., 2020), which asserts that for the moment, it is more useful to simply identify the NC of the AC since this type of consciousness can be studied experimentally while the PC is more elusive. They do consider the PC to be closely linked to the AC, as the subjective experience of the PC constantly accompanies the AC (Dehaene et al., 2001). However, GNWT does not explain the mechanism that produces the PC, “Global Workspace Theory is all about access not about seeing” (Lamme, 2010). As already mentioned, in the continuation of this article I will attempt to describe this mechanism. As originally put forward, the GNWT (Dehaene et al., 2006) supposed that there is a first “preconscious” phase lasting about 200ms, in which there are perceptual processes bottom-up, which are not in the spotlight of attention. In the next conscious phase, top-down attention would select certain perceptions from the unconscious contents to be amplified and thus made conscious. In a recent review on GNWT (Mashour et al., 2020), Dehaene’s group appears to have modified their original theory and now maintain that attention and consciousness are distinct entities. In this modified version, attention acts in the preconscious phase by selecting the contents that will then be made conscious in the next phase of GNW. In this case, consciousness has no function in either selection or attention.

I want to add here that the NC of attention acting in the preconscious phase are probably located in the thalamus (Nakajima et al., 2019; Wolff and Vann, 2019), with selection also requiring input from the ventral striatum and amygdala (Peck and Salzman, 2014; Slagter et al., 2017), based on innate or acquired emotional values (Edelman, 1989; Damasio, 1994; Raymond, 2009). It has been shown that in the preconscious period there is the ability to recognize in the secondary cortices the identity of an object in an unconscious way (Logothetis, 1998; Umiltà, 2000), and even to solve problems of a certain difficulty automatically (Vandenbroucke et al., 2014; Earl, 2019).

If attention on the selected contents remains beyond the 200ms of the preconscious, these perceptions enter the GNW (Mashour et al., 2020). Here, in about 100–150ms, they are amplified (Dehaene and Changeux, 2011) and made conscious. GNW is made up of the most evolved areas of the cerebral cortex. These cortical areas possess long-range axons capable of forming feed-forward and reentrant links that act in a top-down manner with respect to bottom-up processes. This “ignition” would make the selected bottom-up processes aware. In this new format (access consciousness), contents are able to “access” the high-level brain processors which, as mentioned, are responsible for the working memory, the reasoning, the verbal report, decisions, meta cognition, and voluntary control of attention or conscious memories (e.g., semantic, episodic, autobiographical; Baars, 1988, 1997; Dehaene and Changeux, 2004).

According to the group of Dehaene and Changeux 2011, Del Cul et al., 2007, Gaillard et al., 2009, and Mashour et al., 2020, the NC that make up the GNW include the following brain areas: dorsolateral prefrontal (BA 9/10/11/12), anterior dorsal cingulate (BA 32), posterior cingulate-retrosplenial-precuneus (BA 23/7/31), superior temporal lobe (BA 22/37), superior parietal lobe (BA 7a), premotor area BA 6 and premotor BA 8, anterior insula, posterior insula, inferior frontal lobe (BA 44/45/46/47), and inferior parietal lobe (BA 39/40), with greatest importance attached to the dorsolateral prefrontal area. If we carefully examine the works of Dehaene and collaborators (Sergent and Dehaene, 2004; Del Cul et al., 2007; Dehaene and Changeux, 2011), we see that in reality, the dorsolateral prefrontal area is activated about 300–500ms after the presentation of the stimulus. This activation interval called P3b (a late-positive component of event-related potential-ERP) is not clearly connected to the precise moment in which one is aware of a certain perception. Instead, it seems that the prefrontal dorsolateral cortex is active in post-consciousness processes like reporting (Lamme, 2010; Boly et al., 2017; Cohen et al., 2020). On the other hand, the areas of the frontal lobe that are activated in a very specific way in the 100–150ms of conscious perception are undoubtedly the inferior lateral frontal cortex (BA 44/45/46/47) and the premotor areas BA 6 (which includes the supplementary motor area) and BA 8 (which includes the frontal eyes field; Lumer and Rees, 1999; Gross et al., 2004; Del Cul et al., 2007; Gaillard et al., 2009; Dehaene and Changeux, 2011). Also in monkeys, in a no-reporting paradigm, the lateral frontal lobe is activated during conscious perception, but only in the lower convexity and premotor part and not in the dorsolateral prefrontal part (Panagiotaropoulos et al., 2012).

In further support of the hypothesis that the dorsolateral part of frontal cortex is not a NC of the AC, study of the consequences of bilateral lesions of this area (Denes and Pizzamiglio, 1999) shows that the damaged functions of various components of this area are those attributed to the high level processors that as we have seen, are the end users of the contents made conscious by the GWT. Furthermore, in the case of bilateral lesion of the lateral frontal lobe, with respect to patients with impaired ability of conscious interaction, the lower frontal lobe (BA 44/45/46/47) is actually involved and not the dorsal prefrontal lobe (Barceló and Knight, 2002; Stuss and Knight, 2013; Odegaard et al., 2017). As already mentioned and as we will discuss in more detail below, the other high-level processors that are responsible for the memories of conscious contents (e.g., semantics, episodic, and autobiographical) are positioned in the areas of the posterior cingulate-retrosplenial-precuneus complex (Cavanna, 2007). GNWT moreover states, in a generic way, how the NC of the AC have different characteristics and yet act simultaneously and in unison. However, it fails to specifically detail the mechanism through which this occurs and how the NC of the AC makes the selected perceptions conscious. In the continuation of this article, the study of neglect and imagery will allow us to describe this mechanism in more detail and in a novel way.

*Tononi’s group*, on the other hand, tried to identify the NC related to PC (subjective consciousness): “… specific neural correlates of consciousness are the neural mechanisms specifying particular phenomenal contents within consciousness, such as colors, faces, places, or thoughts” (Boly et al., 2017). According to IIT, one becomes conscious when, in relation to a certain perception, the NC of the PC connect with each other in a widespread manner, forming an “integrated” connection network in the cerebral cortex, which allows us to experience a unified whole. At the same time, at each distinct moment we have very specific and differing experiences. Similar to the discussion above regarding the GNWT, the exact mechanism of how the NC of the PC make certain external perceptions or those coming from memory ultimately conscious, is not explained in detail by the IIT. It can reasonably be said that GNWT and IIT do not present enormous differences between them, since both propose a similar, although not identical, way that a broad simultaneous activation of evolved areas of the cerebral cortex is essential to make certain perceptions conscious (Lamme, 2018). What principally differentiates the two theories is, as already mentioned, that GNWT refers to the AC while IIT focuses on the PC.

Furthermore, Tononi and collaborators consider the posterior “hot zone,” composed of the temporo-parietal areas of the cortex, to be essential for consciousness, while not considering the frontal cortex part of the NCC (Koch et al., 2016; Boly et al., 2017). The activity of the frontal cortex is not considered crucial for the specific contents of the PC, but it would be responsible “only” for cerebral activities subsequent to conscious perception. These would include working memory, verbal report of the contents of consciousness or to the motor use of such contents. Tononi’s group, in order to demonstrate that the frontal lobe in its entirety is not included among the NC of the PC, refers in particular to a work by Frässle et al. (2014) and to work of the same group of Tononi (Siclari et al., 2017). In work of Frässle et al. (2014), a group of subjects was tested (through fMRI) who have, alternatively, conscious or not conscious experience of certain images in a passive way (binocular rivalry, Wheatstone, 1838); that is, without having to report verbally or with a motor act relative to their conscious perceptions. The work by Siclari et al. (2017) highlights the brain areas active during REM sleep, while the people tested are dreaming without being awakened, and therefore, there is only a passive conscious perception. Therefore, in both works, only the NC relating to conscious perception are highlighted without there being an overlap of NC relating to actions during post-consciousness reporting. From these studies, it is confirmed that many of the NC of the PC are concentrated in the posterior “hot zone” and in fact, the prefrontal dorsolateral cortex is not active. However, some other frontal areas such as the anterior cingulate, the premotor areas of the inferior frontal lobe (44/45/46/47) and of the pre-Rolandic frontal gyrus (BA 6-8), and the anterior and posterior insula are all active in these situations of passive perception. Therefore, it cannot be completely excluded that the frontal lobe is part of the NCC. However, Tononi’s group has affirmed that bilateral lesioning of the frontal areas does not lead to loss of consciousness, but lesioning of the posterior “hot zone” does (Boly et al., 2017). However, regarding the 10 NCC, only lesioning of the medial parietal lobe (posterior cingulate-retrospenial-precuneus) in the “hot zone” leads to loss of consciousness (Damasio, 1999; Vogt and Laureys, 2005; Cavanna, 2007). This is because this brain area constitutes the complex of memories (motor, emotional, semantic, episodic, and autobiographical; Rolls, 2019), which is a fundamental NC for consciousness. Furthermore, there is also another NCC in the frontal lobe, the anterior dorsal cingulate, whose bilateral lesion involves loss of consciousness (Cairns et al., 1941; Laplane et al., 1981; Damasio, 1999). This latter finding confirms that the anterior dorsal cingulate seems to have the important task of being the activator of the remaining eight NCC for the time necessary (about 100–150ms) for a perception to become conscious. On the other hand, bilateral lesions of each of these eight NCC result in important cognitive deficits but not the loss of consciousness, precisely because there are eight types of parallel and connected functional centers; the lesioning of any one of them is compensated for, even if not completely, by the remaining seven and does not involve total loss of consciousness (Frigato, 2014). Therefore, the fact that bilateral lesions of certain brain areas do not produce complete loss of consciousness does not absolutely exclude the notion that these areas could be NCC as stated by the Tononi group.

In conclusion, by critically comparing the NC of the AC of the Dehaene group (excluding the dorsolateral prefrontal lobe) and those of the PC of the Tononi group (instead including different areas of the frontal lobe), the result is that they actually coincide: anterior dorsal cingulate (BA 32), posterior cingulate-retrosplenial-precuneus (BA 23/7/31), superior temporal lobe (BA 22/37), superior parietal lobe (BA 7a), premotor area BA 6 and premotor BA 8, anterior insula, posterior insula, inferior frontal lobe (BA 44/45/46/47), and inferior parietal lobe (BA 39/40).

The primary sensory areas for vision, including the occipito-temporal sensory cortices BA 17-18-19-20-21 and parietal BA 7b, are certainly involved in conscious perception; however, they are not active exclusively during conscious perception but also in the preconscious phase (Lamme, 2010; Earl, 2014) during which perceptions are still unconscious. Therefore, they cannot be considered specific NC of consciousness. *It can thus be stated that AC and PC have the same 10 NC and therefore, AC and PC are mirror images of each other; they are two faces of the same coin, that is, of the only consciousness in which cognitive and subjective contents are simultaneously present*. From the study of neglect described below, it will be possible to confirm this statement and also demonstrate that the mechanisms generating the AC have the simultaneous consequence of generating the PC.

## A Review of the Literature of the Pathology Called “Neglect”

As already mentioned, neglect is associated with brain damage, generally in the right hemisphere, which specifically prevents the conscious perception of the left hemi-space. Neglect consists of a disorder characterized by the patient’s difficulty in exploring, paying attention, perceiving an act near the body and in extracorporeal space opposite to the injured cerebral hemisphere (Vallar, 1998; Verdon et al., 2010). Therefore, the patient with neglect is unable to bring back the images present in the left space, shaves only the right side of the face and eats only the food present on the right side of the plate (De Renzi, 1982). Only a lesion of the right hemisphere causes severe neglect. One hypothesis for this fact is that the right hemisphere is able to perceive both the left and the right space, while the left hemisphere would perceive only the right space. Therefore, a lesion on the left does not compromise vision on the right since in this case the intact right hemisphere is still able to perceive all the space on its own. On the other hand, a lesion of the right hemisphere prevents the conscious perception on the left since this lesion cannot be compensated for by the intact left hemisphere, which is able to perceive only the right space (Heilman et al., 1987; Lunven and Bartolomeo, 2017). As for the causes of neglect, according to some authors it is due to an attention deficit, while for others it is due to damage to conscious representation. A certain level of agreement has been reached on this point and this pathology is now typically described as being caused from time to time, by disorders of attention, consciousness, or memory (Bisiach and Luzzatti, 1978; Vallar, 1998, 2007; Berti, 2004). It has been shown that the patient with neglect, while stating that he does not see anything in the left space, is able to guess, in an unconscious way, some characteristics of objects placed on his left up to the semantic level, and to solve simple tests (Marshall and Halligan, 1988; Umiltà, 2000). We may interpret these data to show that the patient with neglect, as far as perception in the left space is concerned, is at the preconscious level. Neglect is therefore a pathology in which there is a loss of consciousness, but the ability to process at an unconscious level remains. This allows one to identify in a specific way the NCC for which lesioning causes loss of consciousness. In fact, even lesion of the brainstem causes a loss of consciousness, but it also prevents the unconscious perception, and therefore, the brainstem cannot be considered a specific NCC (Umiltà, 2000). Therefore, identifying the brain areas for which lesioning causes neglect can give us important indications on which are the specific NCC. Furthermore, the study of neglect will allow us to hypothesize that there are eight different types of functional centers with peculiar characteristics and qualities, which act in parallel and independently, and at the same time are closely connected to each other (Frigato, 2014).

## The Neglect Study Confirms the 10 Common NC for AC and PC

There are 10 areas for which lesioning on the right causes left-neglect. Anterior dorsal cingulate (BA 32), posterior cingulate-retrosplenial-precuneus (BA 23/7/31), superior temporal lobe (BA 22/37), superior parietal lobe (BA 7a), premotor area BA 6 and premotor BA 8, anterior insula, posterior insula, inferior frontal lobe (BA 44/45/46/47), and inferior parietal lobe (BA 39/40; Vallar, 1998, 2007; Manes et al., 1999; Cereda et al., 2002; Halligan et al., 2003; Verdon et al., 2010; Lunven and Bartolomeo, 2017). These 10 areas correspond to the NCC common to GNWT and IIT; that is, to the NC of the AC identified by the Dehaene group, excluding the dorsolateral prefrontal lobe, and to those of the PC identified by the Tononi group, including some frontal areas. In fact, in the literature, when there is neglect from lesions of the lateral frontal lobe, in reality it refers to the inferior frontal lobe and premotor BA 6-BA 8 (Husain and Kennard, 1997; Verdon et al., 2010) and not to the dorsolateral prefrontal region. The study of neglect therefore confirms that AC and PC are two faces of the same coin, as had already been suggested by some authors based on psychological-philosophical considerations (Cohen and Dennett, 2011; Overgaard, 2018). We will further explore what consequences this may have for attempting to solve the “hard problem” of the PC.

## Activation and Memories Linked to Consciousness

In neglect, right-side lesioning of any one of the 10 areas is sufficient to cause the loss of consciousness of the entire left part of the space. This would suggest that the bilateral lesion of any single area among these 10 is sufficient to cause total loss of consciousness (Koch, 2004). Koch (2004) therefore examined the consequences of bilateral lesioning of the lateral parietal lobe and found that there was no total loss of consciousness. Such injury caused “only” simultagnosia (Rizzo and Vecera, 2002), that is, the inability to consciously perceive multiple objects together. Koch therefore came to the conclusion, erroneously, that the study of neglect is not useful for identifying NCC. In reality, as already mentioned, only bilateral lesions of the anterior dorsal cingulate (Cairns et al., 1941; Laplane et al., 1981) and of the precuneus-retrosplenial-posterior cingulate complex (Damasio, 1999; Vogt and Laureys, 2005; Cavanna, 2007) result in loss of consciousness and “zombie-like” behavior (Damasio, 1999). This result demonstrates the fundamental role of these two areas (Rolls, 2019) for the production of conscious content. Also in the monkey, bilateral lesioning of the anterior cingulate causes “zombie-like” behavior, confirming Damasio’s observations in humans. In fact, monkeys with this lesion have only automatic behaviors and are not able to perform flexible actions supported by reasoning (Stern and Passingham, 1994; Rushworth et al., 2003), which require the presence of conscious contents. The literature also attributes to the anterior dorsal cingulate (BA 32) an important role in conscious attention and in modulating executive centers (Posner and Dehaene, 1994; Bush, 2011). As for the posterior cingulate-retrosplenial-precuneus complex, it is believed that this is the seat of high-level memories such as semantic, episodic, and autobiographical ones (Vogt and Laureys, 2005; Cavanna and Trimble, 2006; Burianova and Grady, 2007; Cavanna, 2007). It may contain all eight memories of motor (3) –perceptual (4) – and emotional (1) conscious contents. The exact locations of each of these memories have not yet been identified and distinguished one by one. However, there are clues, for example, regarding the posterior cingulate for semantic, motor, and body memory (Rolls, 2019) and the precuneus as the seat of episodic and autobiographical memory (Cavanna and Trimble, 2006).

In summary, it can be assumed that the anterior dorsal cingulate area is responsible for the activation of the NC of the eight functional centers and the posterior-retrosplenial-precuneus cingulate complex is responsible for storing the memories of the corresponding eight conscious contents. These memories provide the ability to relive, through imagery or remembering, the experiences lived with all of their eight perceptual, motor, emotional, and subjective characteristics. Further evidence supporting the importance of the anterior dorsal cingulate and the complex of memories includes the observation that recovery after an injury (vegetative state) or after anesthesia is accompanied by the gradual restoration of the activity of these two brain areas (Rolls, 2019).

## Eight Parallel Functional Centers

If we now examine the consequences of bilateral lesioning of each of the other eight NCC, it is noted that they involve important and specific cognitive deficits but do not cause loss of consciousness. Indeed, the neglect, or the deprivation of consciousness of the left space, is no longer present. For example, bilateral lesioning of the medial-superior temporal area (BA 22/37) “only” causes semantic agnosia (Rogers et al., 2004), i.e., the inability to consciously recognize a particular object, while all of the functions linked to the other seven functional centers are sustained. The patient remains, in fact, aware of his own body, his emotions, his movements, and of himself as an agent, etc (Grossi et al., 1988). In the same way, bilateral lesioning of the superior parietal lobe only (BA 7a) causes, as mentioned, simultagnosia (Rizzo and Vecera, 2002). The environment is not perceived as a whole, but the patient can see one object at a time. Again, there is no complete loss of consciousness and the other seven functions remain intact.

In an attempt to explain these counterintuitive data, recall that the eight functional centers whose single lesion in the right brain causes neglect have eight mirror areas in the left brain. Furthermore, it must be kept in mind that the eight areas, both on the right and on the left, are closely connected by the three branches of the superior longitudinal fasciculus. In fact, neglect can be caused not only by lesion the right side of one of the eight functional centers, but also by lesions to the right of the superior longitudinal fasciculus causing a disconnection syndrome (Bartolomeo, 2007; Verdon et al., 2010; Lunven and Bartolomeo, 2017). Therefore, it can be hypothesized that lesioning the right part of the brain of a single functional center is sufficient to cause neglect since all the other seven centers connected to this are weakened. This in turn, favors the left part with the mirror eight centers intact, which therefore becomes dominant. An imbalance is created and only the right side of the space is therefore perceived and becomes conscious. The fact that when a lesion of one of the eight centers is bilateral, the observed result is that there is “only” a specific deficit connected to that particular center, can be explained by hypothesizing that the eight centers operate in parallel both on the right and on the left, with eight different characteristics and peculiarities. Therefore, when there is a mirror bilateral lesion of a certain functional center, the balance between the two parts of the brain are restored with the disappearance of neglect and without total loss of consciousness (Frigato, 2014). In conclusion, it can be said that the eight centers operate in parallel and at the same time are closely linked to each other.

As stated several times, the distinguishing feature of neglect is the generalized loss of consciousness of the left space. However, a careful and thorough examination of this pathology shows that the lesions to the right of the different brain areas responsible for neglect actually involve different types of neglect with neuropsychological characteristics peculiar to each of these areas (Halligan et al., 2003; Hillis, 2006; Verdon et al., 2010). By adding to these data the identification of their functions through fMRI, the specific functions related to each of the eight areas can be determined.

## Functions of the Eight Functional Centers

The functions of the eight types of centers are predicted as follows:

1. The lesion to the right of the medial-superior temporal lobe (BA 22/37) causes allocentric neglect (Hillis et al., 2005; Chechlacz et al., 2010; Verdon et al., 2010), i.e., the inability to perceive consciously the right side of an object. As already mentioned above, the bilateral lesion of this area causes semantic agnosia, that is, the inability to have the conscious semantic knowledge (“what”) of an object. The fMRI confirms that this area is active both in the perception (Mishkin and Ungerleider, 1982) and in the imagery of objects (Mechelli et al., 2004).
2. The lesion of the right superior parietal lobe (BA 7a) causes the inability to consciously determine the position (“where”) of an object in the left part of the space (Vallar and Perani, 1986; Vallar, 2007). We have seen that the bilateral lesion of BA 7a causes simultagnosia, that is, the inability to be aware of more than one object at a time. It has been confirmed by fMRI that this area is responsible for identifying the position of an object (Mishkin and Ungerleider, 1982).
3. The lesion in the right premotor area (BA 6) which includes the supplementary motor area, causes motor peripersonal neglect, i.e., the inability to perform voluntary motor acts, real or potential, on one’s own body or close to it (Committeri et al., 2007). fMRI confirms these features (Halligan et al., 2003; Verdon et al., 2010).
4. The lesion in the right premotor area (BA 8) which includes the frontal eye fields causes motor extrapersonal neglect, i.e., the inability to perform voluntary movements, real or potential, in the extrapersonal space (Committeri et al., 2007). fMRI confirms these features (Halligan et al., 2003; Verdon et al., 2010).
5. The lesion of the right anterior insula causes anosognosia (Ibañez et al., 2010; Vocat et al., 2010), that is, the inability to consciously realize one’s disability. This result supports one theory, that anosognosia is due to an emotional deficit (Damasio, 1994). The fMRI confirms the involvement of the insula in the conscious emotion (pleasure, pain, fear, etc.) related to a perception (Singer et al., 2004).
6. The lesion of the right posterior insula causes hemisomatoagnosia, that is, the loss of consciousness of the left side of the body. In the most severe cases, there is somatoparaphrenia (Vallar and Ronchi, 2009); the patient is convinced that the injured limb does not belong to him and tries to throw it out of bed (Ibañez et al., 2010). The fMRI shows the activation of the posterior insula in body awareness (Tsakiris et al., 2007).
7. The lesion in the right inferior frontal cortex (BA 44/45/46/47) causes the inability to select, to decide and to pay attention to the most important contents in the left space (Halligan et al., 2003; Verdon et al., 2010; Lunven and Bartolomeo, 2017). The fMRI confirms that we can consider this area responsible for the feeling of being the Author (Brass et al., 2005) in the “theater of consciousness” (Baars, 1997).
8. The lesion of the right inferior parietal lobe (BA 39/40) causes egocentric spatial neglect with the loss of consciousness of one’s position as an observer in the left space (Vallar and Perani, 1986; Lunven and Bartolomeo, 2017). The fMRI confirms the role of this area in the first person perspective or in the imagery of an act (Lou et al., 2004). Its stimulation gives the sensation of floating in space (Blanke et al., 2002). We can consider this area responsible for the feeling of being the Spectator (Grivaz et al., 2017) in the “theater of consciousness.”

## Imagery and the Convergence Mechanism

Given that there are situations, in which the imagery is unconscious (Nanay, 2021), I want to specify that below I will refer explicitly to the conscious imagery. In fact, to try to understand what is the mechanism of action of the eight functional centers, it is important to take into consideration the fact that if we compare the NC of the external visual perception of an object with those of its visual imagery, there is overlap between them (Ganis et al., 2004; Pearson, 2019). Similarly, there is overlap between the NC of motor execution with those of the imagery of motor act (Hanakawa et al., 2003). Obviously there is an activation gradient – first, in the perception of an external object as a source of images, the occipito-temporal sensory cortices are much more active and second, in the imagery, the areas of the posterior-retrosplenial-precuneus cingulate that provide the memory images are much more active (Mechelli et al., 2004; Ishai, 2010). In both situations, eight areas are active in the same way (Ganis et al., 2004; McNorgan, 2012) and they correspond to the eight NCC. These areas are not specifically parts of either of the areas responsible for external perception or memory. Being associative areas, this brings to mind “the points of convergence in the third person” theorized by Damasio (1994) and McNorgan et al. (2011). Briefly, we can say that Damasio affirms that these points or centers of convergence keep active at the same time both the image of an object and the modification of one’s body operated by the emotion connected to that particular image. According to Damasio, this synchronization mechanism between three areas provides perceptual awareness. In my opinion, the mechanism described is concerned with many more types of awareness; it is more distributed, parallel and it involves memory in a fundamental way. In our case, the intelligent mechanism of the third-person convergence centers, as originally conceived by Damasio, can be extended to explain how the cortices responsible for sensory-motor-emotional perceptions can be kept simultaneously connected to their eight corresponding memories. The NC of the eight parallel functional centers of consciousness would each function precisely, as a point of convergence in the third person, connected in a reentrant way both with the respective perceptual cortex and with the corresponding memory. To obtain the external conscious perception, in addition to the image coming from the environment, it would therefore be necessary to simultaneously activate and superimpose the corresponding mirror memory for that image. And vice versa for conscious perception through imagery. Between the neurons responsible for sensory perception and those required for memory, there would have to be continuous feedback with an alternation of the activation gradient between external sensory cortices and the complex of memories.

For example, in the case of the conscious perception of an object (the “what”), the center of convergence medial-superior temporal lobe (BA 22/37) simultaneously keeps active both the inferior occipito-temporal cortex, which is responsible for the external visual perception of the “what,” and the part of the posterior-retrosplenial-precuneus cingulate complex seat of semantic memory. In the case of the conscious perception of the position (the “where”) of an object, the superior parietal lobe NCC (BA 7a) would act as the center of convergence between the neurons that detect the spatial position of the “where” of the object (BA 7b) and the memory neurons of the same or similar positions.

The anterior dorsal cingulate (BA 32) would have the function of keeping the eight convergence centers active for the time necessary, about 100–150ms, for the perception to become conscious and in a format that can be used by the processors of high level. This format is therefore characterized by close connections between the parallel centers of convergence which in turn, ensure that there is a continuous alternation and overlap between the images of the external environment and those of memory.

The anterior dorsal cingulate would keep more active the NC of the conscious content prevalent at that moment among the eight, and to a lesser extent also the NC that control the other seven contents. For example, in the case of the prevailing semantic perception of an object (the “what”), the dorsal anterior cingulate would activate more the center of convergence medial-superior temporal lobe (BA 22/37) and to a much lesser extent and in the background, the other seven centers, which are nonetheless fundamental for the complete conscious perception. This process would result in a single consciousness made up of eight different functionalities. Together they make up the contents of consciousness which are both cognitive and subjective.

Furthermore, each functionality will have specific contents within it, depending on the information that comes from the different sensory cortices in the form of varied shapes, colors, tactile sensations, smells, tastes, sounds, joys, or pains. For example, in the perception of “what,” whether the perception is visual, tactile, auditory, gustatory, or olfactory, they each refer to the same center of semantic convergence in the medial-superior temporal lobe (BA 22/37; McNorgan, 2012). The same applies to the emotions connected to the five senses that all converge in the anterior insula (Brown et al., 2011). The NC that differentiate the conscious perception of one of the five senses from the other are those corresponding to the modality-specific sensory cortices which are, for example, the occipitotemporal ones (BA 17/18/19/20/21) for vision or the postrolandic parietal ones (BA 1/2/3) for touch (McNorgan, 2012).

The speculation of the existence of eight parallel and connected functions that make up consciousness seems to be confirmed by studies showing that when you think, for example, of an apple, the brain areas related to memory of shape, color, taste, and touch are all activated simultaneously with the ones related to our previous experiences with apples (Mitchell et al., 2008; Just et al., 2010). Memories can therefore be semantic, motor (e.g., handling or biting an apple), episodic (e.g., Adam and Eve), or autobiographical (related lived experiences). The areas that are activated are the posterior cingulated, superior temporal cortex, superior parietal, frontal lateral and inferior frontal premotor, insula, and inferior parietal (Just et al., 2010), basically all of the NCC that are responsible for the eight functional aspects of consciousness.

## The Hard Problem of Phenomenal Consciousness: Revisited

If, as mentioned, the NC of the AC and the PC coincide, then the sensations or “qualia” of the PC can be explained as a product of the simultaneous functioning of the eight different centers of convergence. Therefore, one would no longer consider AC and PC as two separate forms of consciousness, but instead they would constitute a single consciousness made up of different types of parallel and closely related contents, along with multiple external perceptions and corresponding memories. Beginning with this concept, we can then attempt to define consciousness as an amalgam of various perceived contents including the following: the experience of perceiving a particular object or event from the environment or memory, its shape and colors, its position in space, its connected emotions, the intention to perform a motor action on that object, the presence of one’s own body, and the feeling of being both the Author and the Spectator of this perception. It is critical to highlight that all eight distinct sensations are directly or indirectly linked to the body of the subject who has conscious perception: the presence of one’s body, emotions (viscera), motor activities (muscles), self-agent (again the muscles), the position of the object in space (with respect to the observer), the Author, and the Spectator.

This definition indicates that consciousness is not only access or cognitive consciousness, it is also indissolubly, phenomenal consciousness or subjective consciousness with a very complex and varied set of sensations. These sensations are difficult to describe as they are the result of the interaction and coordination of eight different centers, along with their associated perceptions and related memories, which ultimately produces a very complex and articulated whole that is absolutely personal-subjective. Therefore, when we have either a direct experience of the external environment or when we rely on mental images, we are operating beyond the cognitive dimensions of typical thought; in a sense, we are operating beyond our usual level of understanding. For this reason, the “hard problem” of the PC seems incomprehensible. Thus, it appears to us evanescent and abstract, devoid of any evident usefulness. It is as if the explanation of PC is something immaterial or spiritual emanating from something material like the brain.

The complex conscious experience is lived and memorized with its cognitive and subjective contents, which can both be simultaneously relived through the imagery. This suggests that the subjective content of qualia is also useful from an evolutionary point of view. For example, it is very important to perceive and also remember-relive, not only that a fruit is simply yellow or green, but also which subjective sensations are given to us by a particular yellow or green color with its many shades, which may be indicative of its edible nature or not. In the same way, it is not enough to perceive and remember only the cognitive details relating to a particular episode that we have experienced or to a specific person that we have known, but it is essential to also remember the subjective experience or person with its associated qualia.

The intuitive sensations present in the background can lead to a more complete understanding of a life experience and allow one to make correct decisions about it (Brown et al., 2011). Intuition is useful both for solving “social” problems and logical-mathematical problems, as suggested by the famous mathematician-physicist Penrose (Damasio, 1994, 1999). So, it is not true that the PC is a useless illusion constructed by the brain (Dennett, 1993). All consciousness in its entirety, AC+PC, is fundamental for our complete knowledge.

## The Evolution of Consciousness: Revisited

For the purpose of brevity, we will consider only a simplified evolutionary “scale” that does not take into account the real, great complexity of animal evolution. The simplified evolutionary scale will be as follows: amphibians originate from fish; from amphibians to reptiles; from reptiles to birds and mammals; from non-anthropomorphic monkeys to anthropomorphic ones; and finally from the latter, to Man.

The presence of consciousness, as stated by many authors, is certainly fundamental and indispensable for the functioning of important mental processes such as working memory, reasoning, decisions, attention guidance, and non-automatic memorization. In fact, in the past it was often assumed that these high-level processors operated consciously (Baars, 1988; Dehaene and Changeux, 2004). However, we illustrated in the introduction that a certain agreement has now been reached in the belief that consciousness has no executive, attentive, or control functions (Libet et al., 1983; Earl, 2014; Oakley and Halligan, 2017; Mashour et al., 2020). The real and only evolutionary advantage provided by the presence of consciousness is to produce content in a format that can be easily used by high-level processors. For example, according to Earl (2014), the biological function of consciousness is exclusively to create information in different forms to be used by a flexible response mechanism to make decisions, plan, and more generally respond in a non-automatic way. According to Oakley and Halligan (2017), conscious contents can be stored in the autobiographical memory or communicated to our neighbors through language, thus having an important role for social interactions.

Without having to ask “what is it like to be a bat?” (Nagel, 1974), to understand in which animal species consciousness is present, it is necessary and sufficient to establish which of them possess the following: anterior dorsal cingulate, the complex of memories, at least some of the eight centers of convergence, the dorsolateral prefrontal lobe for the use of conscious contents, and the hippocampus for their memorization. At the same time, it is also necessary to determine which intelligent behaviors these species have (Bitterman and Mackintosh, 1969; Denton, 2005) that can only be explained by the presence of consciousness. These cognitive abilities all share the capacity to possess and maintain active mental images for problem solving (Denton, 2005). The following paragraphs briefly detail the evolutionary development of these anatomical brain regions, centers of convergence and cognitive abilities from the most primitive fish to humans.

The most primitive fish appear to lack an evolved pallium and hippocampus (Docampo-Seara et al., 2018) which, on the other hand, are both present in more evolved fish (e.g., teleosts; Fabbro et al., 2015). Probably in the most primitive fish, it is the cerebral areas typical of the preconscious (occipito-temporal cortex), where contents are selected and kept active during the 200ms after the presentation of a stimulus for the execution of simple tasks. The most primitive fish, not possessing working memory or imagery, are able to solve problems based only on perceptions, and are not aware that these perceptions are derived directly from the external environment at that precise moment. On the other hand, conscious animals have the ability to use images from working memory or imagery (Denton, 2005).

In more evolved fish, the contents present in the preconscious are made conscious by the evolved pallium and are subsequently stored by the hippocampus in the complex of memories (Pandya et al., 2015). In this manner, an immediate learning is achieved which is profoundly different from the slow and long learning by trial and error of animals without consciousness. Although, the evolved pallium does not have the six-layered structure of the cerebral cortex, it still has a comparable function (Fabbro et al., 2015). We can assume that in the evolved pallium, in addition to the anterior and posterior cingulate, only four centers of convergence are present. These may include the capacity of semantic perception (BA 22/37 “what”), the spatial perception (BA 7a “where”), that of the body (posterior insula) and that of the emotional perception (anterior insula). There must also exist a part of the pallium with functions similar to the prefrontal cortex of mammals, that is, with the ability to solve problems and have complex behaviors that require the presence of conscious contents. The most evolved fish have similar abilities to other vertebrates (Brown, 2015). These brain areas with high levels of cognitive ability have also been demonstrated in amphibians (Liu et al., 2016) and reptiles (Tosches et al., 2018).

The presence of the cerebral cortex in mammals would lead to a fifth convergence center. In birds, there are agglomerations of neurons that seem to have the same functional capacities as the cerebral cortex, combined with “intelligent” behaviors (Fabbro et al., 2015). Probably in birds and mammals, the BA 8 premotor five convergence center is added and is responsible for extrapersonal motor activity. Obviously, at the same time, the volume of the entire prefrontal cortex would increase. Probably in non-anthropomorphic monkeys the number of six centers of convergence, with BA 6 responsible for peripersonal motor activity, would be reached. In anthropomorphic monkeys, the addition of the inferior frontal lobe (BA 44/45/46/47) would give the feeling of being the Author with the capacity of self-recognition in the mirror (Gallup, 1970). And finally, the maximum number of eight centers of convergence would be reached in Man with the presence of the inferior parietal lobe (BA 39-40; Popper and Eccles, 1977), which gives the feeling of being the Spectator of the scene in the theater of consciousness. This results in the consequent ability to provide content used by high-level processors responsible for meta-consciousness, autobiographical memory, and language.

## Conclusion

In this article, referring to the experimental and theoretical works of a large number of authors, as well as to some of my own considerations, I provide a modified vision of consciousness that is summarized in the following passages.

- In the first 200ms, or in the preconscious, everything happens unconsciously. Perceptions from the external environment activate the primary sensory cortices which in the case of vision, is the V1 striated occipital cortex. From here, the information is sent to the secondary sensory cortices of the inferior temporal lobe, where neurons are activated that are responsible for the “what” perceived by any one of the five senses. For example, object-specific neurons are localized in the anterior temporal lobe, face-specific neurons in the fusiform lobe, spatial map neurons of a scene in the parahippocampus, regardless of whether the perception is visual, tactile, auditory, gustatory, or olfactory. All of these perceptions are connected in an innate or acquired way to the centers of emotional evaluation (pleasure, displeasure, fear, hunger, thirst, etc.), located mainly in the ventral striatum (with the nucleus accumbens) and in the amygdala; these areas are the architects of a selection which is then communicated to the thalamus. It, in turn, keeps active in the inferior temporal cortices, the attention on “what” is most important at that moment. At this point, a feedback loop is activated between the neurons of the inferior temporal cortices and of the primary sensory cortices (V1 in the case of sight) specific for that particular “what” that was selected (Lamme, 2010, 2018). In this way, we arrive at the stage of semantic recognition, but always at an unconscious level (Umiltà, 2000). At this moment, the percept can be processed for minor automatic tasks (Vandenbroucke et al., 2014; Earl, 2019), of which the dorsal striatum is responsible.
- If the thalamic attention continues its active state beyond the first 200ms, at this point the NCC come into action. The anterior dorsal cingulate keeps the eight centers operational for 100–150ms. During this discrete interval, they function as areas of convergence in the third person, keeping the sensory-motor-emotional areas and the corresponding memories activated in parallel with a continuous reentrant feed-back. In these 100–150ms, the selected perception is thus made conscious, that is, in a format consisting of eight different specificities with cognitive (AC) and subjective “qualia” (PC) contents, which together constitute a single entity. This format is available to be used by high-level processors that are capable of operating only with conscious contents and not with unconscious ones.
- The high-level (unconscious) processors of voluntary attention, working memory, decision-making, execution, or control, are mainly located in the dorsolateral prefrontal lobe (BA 9/10/11/12). They precisely process the incoming conscious contents; the unconscious results of the processing are in turn made conscious by the NCC and available again for high-level processors and so on. Thus, a continuous stream of consciousness ensues (James, 1906).
- The hippocampus stores conscious contents.
- The dorsal striatum is responsible for automatic behaviors.

In summary, I have attempted to reconcile two theories of consciousness that are based on either cognitive (AC) or subjective (PC) contents, GNWT, or IIT, respectively. A careful examination of these two theories leads to the conclusion that the NC of AC and PC coincide and in fact, the two consciences are mirror images of each other; they are two faces of the same coin, that is, of the only consciousness, in which cognitive and subjective contents are simultaneously present. New findings from recent studies on attention (Davidson et al., 2020) and consciousness (Mashour et al., 2020) are converging to validate the hypotheses set out in this article. We hope this trend continues.

## Data Availability Statement

The original contributions presented in the study are included in the article/supplementary material, further inquiries can be directed to the corresponding author.

## Author Contributions

The author confirms being the sole contributor of this work and has approved it for publication.

## Conflict of Interest

The author declares that the research was conducted in the absence of any commercial or financial relationships that could be construed as a potential conflict of interest.

## Publisher’s Note

All claims expressed in this article are solely those of the authors and do not necessarily represent those of their affiliated organizations, or those of the publisher, the editors and the reviewers. Any product that may be evaluated in this article, or claim that may be made by its manufacturer, is not guaranteed or endorsed by the publisher.
